# Ian Munro – father of synchrotron radiation in the UK (1937–2022)

**DOI:** 10.1107/S1600577522011924

**Published:** 2023-01-01

**Authors:** S. Samar Hasnain

**Affiliations:** a Institute of Systems, Molecular and Integrative Biology, University of Liverpool, Life Sciences Building, Crown Street, Liverpool L69 7ZB, United Kingdom

**Keywords:** obituary

## Abstract

Obituary for Ian Munro.

It was Sunday afternoon, 29 September 1974, when I arrived at Manchester Airport, the year in which the ‘air bridges’ connecting passengers direct from the aircraft to the terminal had become operational at Manchester. A smiling face full of welcoming enthusiasm, so typical of Ian Munro, recognized me and came forward with his positive style so that I did not have to wonder or worry if there would be someone there to meet me. I soon discovered that I was not going to the student accommodation but to his home, where I stayed for a whole week. I was moving from my mother’s home in Karachi to Ian’s home in Didsbury – both full of love – a home from home.

[Chem scheme1] 
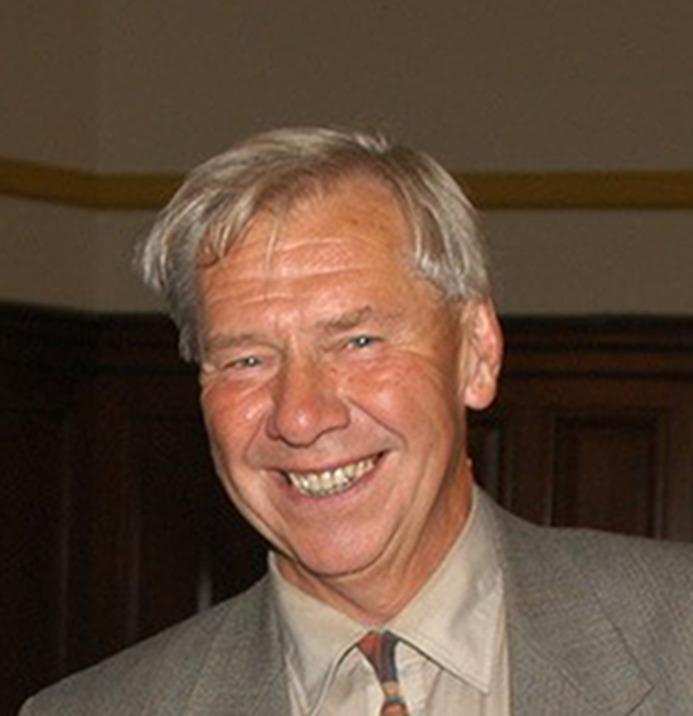


During the daytime through the week, he devoted a lot of time introducing me to the department colleagues and the tea room, helping sort out my accommodation in Owens Park and opening my bank account (almost 50 years later, I still have the same account). In the evening, we would travel from the university to home in his VW van, where he and Joan would rustle up dinner for the four kids (Robert, Elizabeth, Neil and Janet) and me. What a man! Caring, warm, loving and, above all, a modern family man, sharing duties of the kitchen and everything in the days when it was not the norm.

During the next three years, initially as a PhD student, then as a PDRA, I joined Ian Munro in his Synchrotron Light Project, which he had established in 1973 at the Daresbury Laboratory, in the village of ‘Alice in Wonderland’. He had taken a bold action upon his return from a sabbatical in Canada to write a letter to the Director of Daresbury, Alec Merrison, in 1967, to obtain his support to explore the properties and use of light from an electron synchrotron that had been commissioned in1967 for a different scientific purpose (particle physics). His vision, enthusiasm, ability to bring people together and endless energy resulted in him obtaining twice the funding he had requested, allowing him to establish two beamlines rather than just one. When I arrived in 1974, there were already teams from Reading, Oxford, Bristol and the famous MRC Laboratory of Molecular Biology in Cambridge. Teams from Leicester and Warwick soon joined this amazing project.

While we were immersed in the new exciting science made possible by this new tool, Ian, with other senior science leaders, was working on a way to get a more ambitious project approved, as he knew that the parasitic synchrotron light facility was going to close in a few years. In 1976, we learned that the closure would be sooner than even Ian had expected only a couple of years before. It happened on 1 April 1977 with the highly positive news that the UK’s Government Science Research Council had decided to build the world’s first dedicated purpose-built Synchrotron Light Source (later to be known as the SRS) at Daresbury. On 31 March 1977, he worked with the troops until late in the evening before returning home to Didsbury. The next morning, we had a closing ceremony for the old NINA facility and the announcement of the start of the construction phase of the SRS. Ian duly turned up, enthused, and thanked the troops and made us all happy. It was only later that we discovered that Ian had spent the rest of the late evening of 31 March with his father, who lived a few doors away from his home. His dad had passed away during the night as if he knew the importance of Ian’s presence at Daresbury on 1 April 1977 at 11 am. Ian was an exemplary leader. ▸

After the closure of NINA, Ian took the opportunity to spend a year at the Stanford Synchrotron Radiation Project (SSRP, but now SSRL) in California from September 1977. Here, Ian set up a new experimental facility through his ingenuity and persuasion in a Sears garden shed (nothing fancy but a world’s first) installed on the roof of the Stanford Positron Electron Accelerating Ring (SPEAR), and published a seminal paper (Munro *et al.*, 1979 ▸) with the world-renowned biochemist Lubert Stryer.

In September 1978, he returned to Daresbury, working to make the SRS – which became operational in 1980 – not only the world’s first but also the leading light to the world’s scientists. Many leading researchers from across the world became regular visitors and friends of Ian, Daresbury and the UK. Two of these collaborations are worth mentioning: one with the Soviet Union through the Royal Society agreement with the Academy of Sciences, and the other between the Science Research Council (Daresbury Laboratory was one of their laboratories) and the Japanese Ministry of Education, Science and Culture. The second of these led to a close relationship with the Institute for Molecular Science in Okazaki.

Many of the researchers in the UK benefited from his vision, enthusiasm and support. Several became Fellows of the Royal Society as a result of their work at the SRS. Before his retirement, the SRS achieved another world’s first, the first Nobel Prize given to work based on synchrotron X-rays, to Sir John Walker, marked by *Journal of Synchrotron Radiation* through a special issue on Synchrotron Radiation and Structural Biology in July 1999 (Volume 6, Part 4).

There are many stories that I can tell, but here I offer only three short ones. During the NINA SRF operation, Ian would always come back in the evening whenever we had a dedicated time. Quite often, he would make coffee/tea from the office building located 100 yards from the experimental hall and bring several cups balanced on a tray. One wet evening, he came into the experimental hall and saw some frogs. His ‘save the frogs’ instinct prompted him to immediately open the big shutter doors designed to receive large equipment and let the frogs out. Later on, Ian was horrified to discover that these were special frogs that had been imported from Hungary by Hugh Huxley and Wasi Faruqi for the very important work of understanding how muscles work (Huxley & Holmes, 1997 ▸).

Another story relates to an incident that occurred on the South Beamline of the NINA SRF, where numerous exploratory X-ray experiments were conducted, which often required new installations and changeover. It was the beamline where muscle diffraction had a permanent installation, whereas others such as protein crystallography, white-beam topography and energy-dispersive powder diffraction experiments required putting things together for a particular experiment, which sometimes called for a hectic pace of work. Ian would join in helping to assemble the components, partially to see that the exploratory experiments got the best chance but also to share in the excitement of new results from these experiments. During one such exercise, technicians were working on some installation on the mezzanine floor while the scientist and engineers were putting things together for an experiment using an Li-drifted solid-state detector for the first time on a synchrotron (Bordas *et al.*, 1976 ▸), going in and out of the southern experimental hall. To try out the experiments with the beam, they searched the area and locked up the hall, soon to discover an electrician running out and breaking the emergency exit – he had been working on the mezzanine, which Ian and his colleagues had forgotten to check.

The final story relates to a visit by my group in February 1986 to the Photon Factory in Japan. Ian was visiting another laboratory in Japan in Okazaki at the time and decided to come to the Photon Factory in Tsukuba, whose director at the time decided to throw a party in Ian’s honour. To our surprise, it followed karaoke, which was very fashionable in Japan then, but we had not yet come across it in the UK. As we were very few from the UK compared with the Japanese contingent, we were joined by a couple of senior visitors from Germany. The Japanese party and us had to alternate. Initially, we did pretty well, but soon we started running out of material. Ian, thinking on his feet, suggested singing God Save the Queen – the German colleagues joined in happily. We could finish the party with our heads held high.

On a personal note, his return to Daresbury in 1978 was good fortune for me. I had gone to Germany in September 1977 when Ian had gone to sunny California. I came to the UK in September 1978 en route to emigrating to the USA. Ian persuaded me to stay in the UK and take up one of the job offers there. I took up a PDRA position in Manchester Chemistry and Medical Biophysics departments with David Garner and David Hukins – the rest is history – all of which is owed to Ian Munro sharing lots of joyful time, whether experiencing getting a tailor-made suit for Ian (who normally did not wear a suit) or getting a special tailor-made wedding dress for his second wife, Caroline, with the master tailor doing the fitting trial in the comfort of her hotel room. It has been the greatest privilege of my life to know Ian Munro and to be able to call him my true mentor and friend. Thank you, Ian, for looking after me and many others who came across you. Ian – you are rightly regarded as the Father of Synchrotron Radiation in the UK and will be remembered so for many years to come.

Ian Munro was a founding co-editor of *Journal of Synchrotron Radiation* and helped the author during the establishment phase of the journal through advice and support. A fuller version of this obituary, with contributions and recollections from more of Ian’s colleagues, is available in the latest issue of the *IUCr Newsletter* (Vol. 30, No. 4).

## Figures and Tables

**Figure 1:**
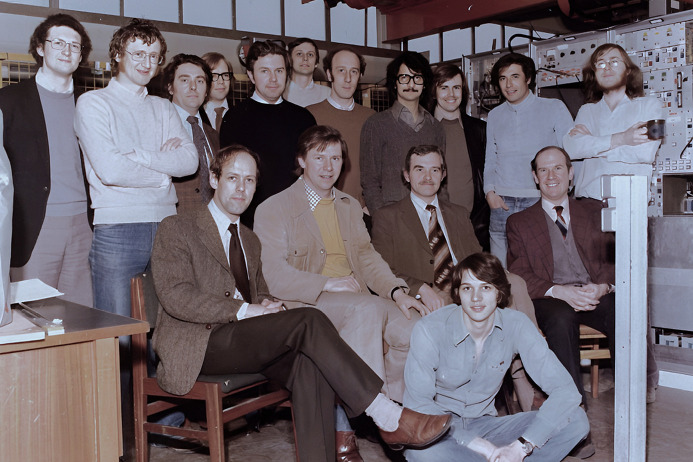
A photograph of the NINA Synchrotron Radiation Facility team taken a few hours before the final switch off of NINA on 31 March 1977. From left to right: Pat Ridley, Iggy McGovern, Bill Smith, Tony Bourdillon, John West, John Beaumont, John Morton, Ian Munro, Paul Brint, Samar Hasnain, Jeff Worgan, Robert Pettifer, Tom Short, Joan Bordas, Ken Lea and Tony Cox.
